# Importance of psychological factors for the recovery from a first episode of acute non-specific neck pain - a longitudinal observational study

**DOI:** 10.1186/s12998-016-0090-2

**Published:** 2016-03-16

**Authors:** Brigitte Wirth, B. Kim Humphreys, Cynthia Peterson

**Affiliations:** Chiropractic Medicine Department, Faculty of Medicine, University of Zurich and University Hospital Balgrist, Forchstr. 340, CH-8008 Zurich, Switzerland; Radiology Department, University Hospital Balgrist, Forchstr. 340, 8008 Zurich, Switzerland

**Keywords:** Acute, Neck pain, Psychological factors, Recovery

## Abstract

**Background:**

The influence of psychological factors on acute neck pain is sparsely studied. In a secondary analysis of prospectively collected data, this study investigated how several psychological factors develop in the first three months of acute neck pain and how these factors influence self-perceived recovery.

**Methods:**

Patients were recruited in various chiropractic practices throughout Switzerland between 2010 and 2014. The follow-up telephone interviews were conducted for all patients by research assistants in the coordinating university hospital following a standardized procedure. The population of this study consisted of 103 patients (68 female; mean age = 38.3 ± 13.8 years) with a first episode of acute (<4 weeks) neck pain. Prior to the first treatment, the patients filled in the Bournemouth Questionnaire (BQ). One week and 1 and 3 months later, they completed the BQ again along with the Patient Global Impression of Change (PGIC). The temporal development (repeated measure ANOVA) of the BQ questions 4 (anxiety), 5 (depression), 6 (fear-avoidance) and 7 (pain locus of control) as well as the influence of these scores on the PGIC were investigated (binary logistic regression analyses, receiver operating curves (ROC)).

**Results:**

All psychological parameters showed significant reduction within the first month. The parameter ‘anxiety’ was associated with outcome at 1 and 3 months (*p* = 0.013, R^2^ = 0.40 and *p* = 0.039, R^2^ = 0.63, respectively). Baseline depression (*p* = 0.037, R^2^ = 0.21), but not baseline anxiety, was a predictor for poor outcome. A high reduction in anxiety within the first month was a significant predictor for favorable outcome after 1 month (*p* < 0.001; R^2^ = 0.57).

**Conclusions:**

Psychological factors emerged from this study as relevant in the early phase of acute neck pain. Particularly persistent anxiety and depression at baseline might be risk factors for a transition to chronic pain that should be addressed in the early management of neck pain patients.

## Background

Neck pain is one of the leading causes for global years lived with a disability [1]. In the general population, its 12 months prevalence ranges from 4.8 to 79.5 % (mean 25.8 %) [2]. Its course is typically fluctuating, but the majority of patients do not completely recover from their symptoms [3] and about 5–10 % of all neck problems become chronic [4].

It is widely established that psychological factors play an important role in chronic non-specific neck pain. Particularly anxiety, depression and catastrophizing seem to negatively affect pain intensity and disability in this patient group [5]. Although different psychological variables might be crucial at different time points in the course of neck pain, patient populations are often rather heterogeneous in terms of symptom duration [6], and only very little is known about this temporal aspect [7]. In patients with sub-acute (and chronic) neck pain, coping strategies that involved self-assurance resulted in better disability outcomes after 6 months [8], while fear of movement hindered short-term (3 months) and long-term (12 months) outcome of sub-acute neck pain as assessed by global perceived recovery, pain and disability [4]. Prognostic factors in acute neck pain are widely investigated in whiplash, but studies in acute non-specific neck pain are sparse. An overview of systematic reviews on prognostic factors for the outcome of a current neck pain episode [9] found two reviews that addressed non-specific neck pain. These reviews [10, 11] revealed two studies that included psychological factors [12, 13]. Bot et al. studied patients with a new episode of neck and shoulder symptoms in general practice and found that pain intensity at baseline, the duration of symptoms before seeking health care, a history of previous neck or shoulder symptoms, reduced vitality and more resting negatively affected self-perceived outcome after 3 months [12]. After 12 months, also more worrying and multiple musculoskeletal symptoms hindered recovery. Hill et al. investigated patients with neck pain in the last month [13]. The strongest risk factor for persistent neck pain after 12 months was age. Further main risk factors were mainly not working at the time of baseline, comorbid low back pain, but also poor general and psychological health were significantly associated with pain persistence. Thus, there is little data available on the impact of psychological factors in the early phase of a non-specific neck pain episode. This might be the reason why Walton et al. concluded in their overview of systematic reviews that in non-whiplash-related neck pain, only older age and other musculoskeletal disorders could be regarded as risk factors for poor recovery, while inconsistent results existed for pain intensity at baseline [14]. The outcome parameters of most studies on psychological risk factors for neck pain were either pain intensity, disability or return to work [7]. However, global ratings of change such as the ‘Patient global impression of change’ (PGIC), which allow the patient to integrate different aspects into one single rating [15] were shown to be more sensitive and to correlate better with the patient’s satisfaction than serial assessments such as pain rating by a visual analogue scale [16]. The above mentioned studies by Bot et al. and Hill et al. assessed global recovery, but used non-validated recovery measures [12, 13].

Thus, in order to prevent acute neck pain from becoming chronic, the goals of this study were to investigate how psychological factors (anxiety, depression, fear avoidance, health locus of control) develop in the first 3 months after a first episode of acute neck pain, and how these psychological factors are associated with self-perceived recovery (assessed by PGIC). We hypothesized that i) the investigated psychological variables decreased in the first 3 months, ii) high psychological distress co-occurred with poor outcome, iii) high scores in the psychological variables at baseline were predictive for poor outcome, and iv) reduction in psychological distress led to favorable outcome.

## Methods

### Participants

This study is based on the secondary analysis of data that were prospectively collected between 2010 and 2014 [17]. For the prospective cohort study with 1 year follow-up, neck pain patients over 18 years with pain of any duration were recruited from various chiropractic practices in Switzerland. Patients with specific pathologies that are contraindications for chiropractic treatment (e.g. tumors, infections) were not included. In total, 850 patients were recruited. For the present observational study that focused on acute non-specific neck pain, only patients who reported that they had no previous episode of neck pain and whose present pain episode lasted for less than 4 weeks were included. Whiplash and any signs of radiculopathy were exclusion criteria. These rather rigid criteria were chosen in order to minimize bias by previous history of neck complaints and duration of symptoms. Thus, the sample of this study consisted of 103 patients (68 female; mean age = 38.3 ± 13.8 years) suffering from the first episode of acute, non-specific neck pain (Table 1).

**Table 1:** Characteristics of the study population

	All patients (*N* = 103)	Patients with improvement after 1 month (*N* = 70)	Patients without improvement after 1 month (*N* = 14)	Patients with improvement after 3 months (*N* = 71)	Patients without improvement after 3 months (*N* = 11)
Age (SD)	38.3 (±13.8)	37.5 (±13.8)	47.5 (±15.0)	38.6 (±13.8)	47.3 (±16.2)
Gender (m/f)	35/68	22/48	4/10	27/44	2/9
Pain at baseline (SD)	6.4 (±1.9)	6.5 (±1.8)	5.5 (±2.1)	6.2 (±2.0)	7.2 (±1.7)
BQ 4: anxiety BL/1 m/3 m (SD)	5.5 (±2.9)	5.6 (±2.8)	4.3 (±2.9)	5.3 (±3.0)	6.7 (±1.7)
	2.1 (±2.7)	1.6 (±2.2)	5.0 (±2.9)	1.7 (±2.4)	5.3 (±2.7)
	1.7 (±2.6)	1.4 (±2.2)	2.5 (±2.9)	1.0 (±1.9)	6.0 (±2.0)
BQ 5: depression BL/1 m/3 m (SD)	3.6 (±3.2)	3.5 (±3.2)	3.1 (±2.9)	3.1 (±3.2)	5.9 (±2.3)
	1.6 (±2.7)	1.0 (±2.1)	3.9 (±3.6)	1.2 (±2.3)	4.3 (±3.5)
	0.8 (±2.0)	0.5 (±1.2)	1.9 (±3.0)	0.4 (±1.3)	3.7 (±2.9)
BQ 6: fear avoidance BL/1 m/3 m (SD)	4.7 (±3.1)	4.6 (±3.1)	4.6 (±2.6)	4.5 (±3.2)	5.7 (±1.6)
	2.0 (±2.7)	1.5 (±2.1)	4.4 (±3.8)	1.6 (±2.5)	4.7 (±2.5)
	1.7 (±2.6)	1.5 (±2.5)	2.3 (±3.0)	1.2 (±2.3)	5.2 (±2.4)
BQ 7: locus of control BL/1 m/3 m (SD)	5.0 (±2.8)	5.2 (±3.0)	4.0 (±2.4)	5.0 (±3.0)	4.5 (±1.7)
	2.8 (±3.3)	2.5 (±3.3)	3.9 (±3.4)	2.6 (±3.3)	5.0 (±2.9)
	2.2 (±2.9)	1.6 (±2.3)	4.2 (±4.0)	1.6 (±2.6)	5.7 (±2.5)

*BQ* Bournemouth questionnaire

*BL* baseline

*f* female

*m* male

*SD* standard deviation

*1 m* 1 month

*3 m* 3 months

### Ethics and consent

Ethical approval was obtained from the ethics committee from the Canton of Zurich, Switzerland (EK-19/2009) and all participants gave written informed consent prior to participation.

### Baseline data and outcome measures

Immediately prior to the first treatment, a numerical rating scale (NRS) for neck pain and a separate NRS (0 = no pain, 10 = worst pain imaginable) for arm pain were filled in by the patients. Furthermore, they answered a validated German version of the Bournemouth questionnaire (BQ) for neck pain [18–20]. The BQ is a valid and reliable outcome measure that considers the multidimensionality of musculoskeletal pain. It covers seven dimensions of the bio-psycho-social pain model: 1) pain, 2) disability (activities of daily living), 3) disability (social activities), 4) anxiety, 5) depression, 6) fear-avoidance (work-related) and 7) pain locus of control. External validity of every single item was shown by significant correlations to its established counterpart external measure [18]. At 1 week, 1 month and 3 months after the first consultation, the same data were collected in a short telephone interview that was conducted for all patients by research assistants in the coordinating university hospital following a standardized procedure. In these consecutive assessments, a German version of the PGIC was also presented to the patients. The PGIC is a retrospective seven-point Likert scale that asks the patients how they feel now compared to before the onset of treatment [16]. Its extreme scores are “much better” and “much worse”, respectively.

### Data analysis and statistics

The BQ questions 4 (anxiety), 5 (depression), 6 (fear-avoidance) and 7 (pain locus of control) were entered into the models as independent variables and were analyzed as continuous data. As for the PGIC, only the two scores “much better” and “better”, but not “somewhat better”, were considered a clinically significant change [21]. Thus, the PGIC data were analyzed as binomial data (0 = not improved, 1 = improved). To determine the development of the psychological factors over time, a repeated measure ANOVA was conducted for each of the four BQ questions. In the posthoc tests (Bonferroni), only the differences between two consecutive time points were of interest. To investigate the importance of the psychological factors for self-perceived recovery, a series of logistic regression analyses with the PGIC as dependent variable was conducted in order to avoid over-fitting the models [22]. Into a first model (model 1), only the psychological variables were entered as independent variables. Thereby, to assess the co-occurrence of these factors with self-perceived recovery, the BQ questions at each concurrent time point were used. To determine their predictive value, the BQ questions at baseline were entered into the model. Lastly, to study the impact of changes in these factors on recovery, the changes in the BQ questions (value of baseline – value of the concurrent time point) were used as independent variables. Then, a further logistic regression model (model 2) was run to estimate the importance of the findings in the context of the literature. This model included the significant factors, if any, emerging from model 1, together with age and pain intensity at baseline [12, 14]. Lastly, the receiver operating curve (ROC) was calculated and the area under the curve (AUC) was determined as a measure for accuracy in discriminating between patients who reported clinically significant improvement and the rest. To test for multicollinearity, we calculated the tolerance and variance inflation factor (VIF) values by running a linear regression analysis with the same outcome and predictors, as recommended by Field [23] (p. 297). According to Field [23] (p. 224), we regarded VIF values >10 and tolerance values <0.1 as critical. No multicollinearity was detected (Tables. 2, 3 and 4). Only complete data sets were included in the regression analyses (complete-case analysis). For all other analyses, data sets with missing values were excluded from the corresponding analyses only (available case analysis). The significance level α was set at 0.05 for all analyses. All analyses used IBM SPSS Statistics 21.0 (SPSS, Chicago, IL, USA).

**Table 2:** Co-occurrence of psychological factors with self-perceived recovery

	B (SE)	Exp B (Odds Ratio)	95 % CI Exp B	*p*
**PGIC 1 week (** ***N*** ** = 76):**				
Nagelkerke R^2^ = 0.05; AUC = 0.62 (95 % CI 0.48–0.75; *p* = 0.126)				
BQ4: anxiety	−0.18 (0.13)	0.83	0.64–1.08	0.171
BQ5: depression	−0.01 (0.11)	0.99	0.80–1.23	0.901
BQ6: fear avoidance	0.06 (0.13)	1.06	0.82–1.36	0.667
BQ7: locus of control	0.04 (0.10)	1.04	0.86–1.25	0.717
**PGIC 1 month (** ***N*** ** = 82):**				
Nagelkerke R^2^ = 0.40; AUC = 0.85 (95 % CI 0.73–0.97; *p* < 0.001)				
BQ4: anxiety	−0.55 (0.22)	**0.58**	0.38–0.89	**0.013**
BQ5: depression	0.13 (0.21)	1.14	0.76–1.71	0.540
BQ6: fear avoidance	−0.25 (0.16)	0.78	0.57–1.06	0.110
BQ7: locus of control	0.17 (0.16)	1.19	0.87–1.63	0.286
**PGIC 3 months (** ***N*** ** = 77):**				
Nagelkerke R^2^ = 0.63; AUC = 0.98 (95 % CI 0.94–1.00; *p* < 0.001)				
BQ4: anxiety	−0.60 (0.29)	**0.55**	0.31–0.97	**0.039**
BQ5: depression	−0.13 (0.23)	0.88	0.56–1.39	0.579
BQ6: fear avoidance	−0.17 (0.18)	0.84	0.59–1.21	0.353
BQ7: locus of control	−0.20 (0.20)	0.82	0.55–1.22	0.325

Logistic regressions with PGIC (0 = not improved, 1 = improved) of each time point as dependent variable and the psychological factors of the same time point as independent variables. Multicollinearity diagnostics: Tolerance: 0.27-0.72, VIF: 1.38-3.65. Numbers in bold indicate significant results.

*AUC* area under the receiver operating curve

*BQ* Bournemouth questionnaire

*PGIC* patient global impression of change

**Table 3:** Prediction of self-perceived recovery by psychological factors at baseline

	B (SE)	Exp B (Odds Ratio)	95 % CI Exp B	p
**Model 1: psychological factors as independent variables**				
**PGIC 1 week (** ***N*** ** = 77):**				
Nagelkerke R^2^ = 0.02; AUC = 0.58 (95 % CI 0.43–0.72; *p* = 0.321)				
BQ4: anxiety	−0.09 (0.15)	0.92	0.68–1.24	0.572
BQ5: depression	0.07 (0.12)	1.07	0.85–1.35	0.579
BQ6: fear avoidance	−0.01 (0.12)	0.99	0.78–1.25	0.928
BQ7: locus of control	0.09 (0.12)	1.09	0.87–1.36	0.459
**PGIC 1 month (** ***N*** ** = 82):**				
Nagelkerke R^2^ = 0.17; AUC = 0.76 (95 % CI 0.62–0.90; *p* = 0.002)				
BQ4: anxiety	0.44 (0.21)	**1.55**	1.04–2.32	**0.033**
BQ5: depression	−0.18 (0.16)	0.83	0.61–1.14	0.247
BQ6: fear avoidance	−0.33 (0.18)	0.72	0.51–1.02	0.061
BQ7: locus of control	0.25 (0.16)	1.28	0.93–1.77	0.529
**PGIC 3 months (** ***N*** ** = 80):**				
Nagelkerke R^2^ = 0.22; AUC= 0.83 (95 % CI 0.72–0.93; *p* = 0.001)				
BQ4: anxiety	0.14 (0.22)	1.15	0.74–1.78	0.529
BQ5: depression	−0.41 (0.21)	**0.67**	0.45–1.00	**0.049**
BQ6: fear avoidance	−0.09 (0.18)	0.91	0.64–1.29	0.597
BQ7: locus of control	0.25 (0.18)	1.28	0.91–1.81	0.163
**Model 2: signifiant factors of model 1 and age and baseline pain as independent variables**				
**PGIC 1 week (** ***N*** ** = 77):**				
Nagelkerke R^2^ = 0.001; AUC= 0.52 (95 % CI 0.39–0.66; *p* = 0.763				
Age	−0.001 (0.02)	1.00	0.96–1.04	0.938
Pain at baseline	0.03 (0.14)	0.97	0.74–1.28	0.841
**PGIC 1 month (** ***N*** ** = 83):**				
Nagelkerke R^2^ = 0.18; AUC= 0.74 (95 % CI 0.58–0.90; *p* = 0.005)				
BQ 4: anxiety	0.12 (0.12)	1.12	0.89–1.41	0.321
Age	−0.05 (0.02)	**0.95**	0.91–0.99	**0.021**
Pain at baseline	0.20 (0.16)	1.22	0.89–1.68	0.225
**PGIC 3 months (** ***N*** ** = 81):**				
Nagelkerke R^2^ = 0.21; AUC= 0.80 (95 % CI 0.69–0.91; *p* = 0.002)				
BQ 5: depression	−0.26 (0.12)	**0.77**	0.61–0.98	**0.037**
Age	−0.04 (0.03)	0.97	0.92–1.02	0.163
Pain at baseline	−0.13 (0.20)	0.88	0.60–1.31	0.536

Logistic regressions with PGIC (0 = not improved, 1 = improved) of each time point as dependent variable and the psychological factors at baseline as independent variables. Multicollinearity diagnostics model 1/model 2: Tolerance: 0.39-0.76/0.89-1.00, VIF: 1.31-2.59/1.00-1.13. Numbers in bold indicate significant results.

*AUC* area under the receiver operating curve

*BQ* Bournemouth questionnaire

*PGIC* patient global impression of change

**Table 4:** Prediction of self-perceived recovery by changes in psychological factors

	B (SE)	Exp B (Odds Ratio)	95 % CI Exp B	*p*
**Model 1: psychological factors as independent variables**				
**PGIC 1 week (** ***N*** ** = 75):**				
Nagelkerke R^2^ = 0.05; AUC = 0.62 (95 % CI 0.48–0.76; *p* = 0.119)				
BQ4: anxiety	0.07 (0.10)	1.07	0.88–1.29	0.488
BQ5: depression	0.16 (0.13)	1.17	0.90–1.53	0.235
BQ6: fear avoidance	−0.06 (0.11)	0.95	0.76–1.17	0.603
BQ7: locus of control	0.02 (0.08)	1.02	0.87–1.20	0.806
**PGIC 1 month (** ***N*** ** = 80):**				
Nagelkerke R^2^ = 0.71; AUC = 0.97 (95 % CI 0.94–1.00; *p* < 0.001)				
BQ4: anxiety	0.93 (0.31)	**2.53**	1.37–4.67	**0.003**
BQ5: depression	0.44 (0.36)	1.56	0.77–3.15	0.218
BQ6: fear avoidance	0.35 (0.28)	1.42	0.83–2.45	0.204
BQ7: locus of control	0.09 (0.16)	1.09	0.79–1.50	0.598
**PGIC 3 months (** ***N*** ** = 75):**				
Nagelkerke R^2^ = 0.36; AUC = 0.90 (95 % CI 0.82–0.98; *p* < 0.001)				
BQ4: anxiety	0.30 (0.20)	1.35	0.92–1.99	0.123
BQ5: depression	−0.17 (0.18)	0.85	0.85 0.60–1.20	0.352
BQ6: fear avoidance	0.02 (0.15)	1.02	0.76–1.37	0.074
BQ7: locus of control	0.23 (0.13)	1.26	0.98–1.63	0.163
**Model 2: signifiant factors of model 1 and age and baseline pain as independent variables**				
**PGIC 1 week (** ***N*** ** = 77):**				
Nagelkerke R^2^ = 0.001; AUC = 0.52 (95 % CI 0.39–0.66; *p* = 0.763				
Age	−0.001 (0.02)	1.00	1.44–3.54	<0.001
Pain at baseline	0.03 (0.14)	0.97	0.74–1.28	0.841
**PGIC 1 month (** ***N*** ** = 83):**				
Nagelkerke R^2^ = 0.57; AUC = 0.92 (95 % CI 0.85–0.99; *p* < 0.001)				
BQ 4: anxiety	0.81 (0.23)	**2.26**	1.44–3.54	**<0.001**
Age	−0.01 (0.03)	1.00	0.95–1.05	0.851
Pain at baseline	0.16 (0.21)	1.17	0.77–1.78	0.454
**PGIC 3 months (** ***N*** ** = 82):**				
Nagelkerke R^2^ = 0.12; AUC = 0.75 (95 % CI 0.63–0.86; *p* = 0.009)				
Age	−0.04 (0.02)	0.96	0.92–1.01	0.111
Pain at baseline	−0.27 (0.19)	0.76	0.53–1.10	0.152

Logistic regressions with PGIC (0 = not improved, 1 = improved) of each time point as dependent variable and the *changes* in the psychological factors as independent variables. Multicollinearity diagnostics model 1/model 2: Tolerance: 0.42-0.83/0.85-0.97, VIF: 1.20-2.38/1.00-1.18. Numbers in bold indicate significant results.

*AUC* area under the receiver operating curve

*BQ* Bournemouth questionnaire

*PGIC* patient global impression of change

## Results

One week after the first consultation, 75.6 % (25 missing values) of the patients reported clinically significant improvement. The percentage increased to 83.3 % (19 missing values) after 1 month and to 86.6 % (21 missing values) after 3 months.

**Fig. 1:**
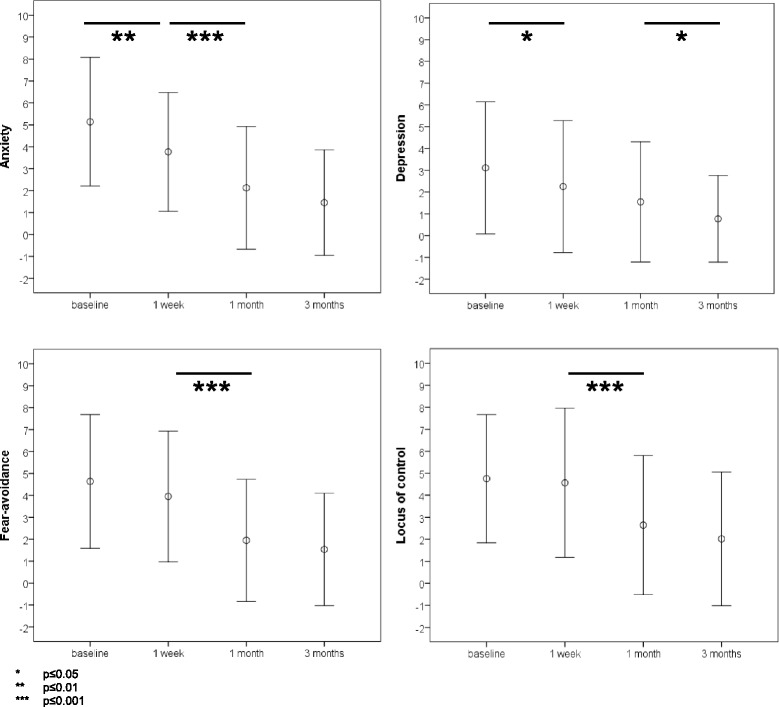
Changes in psychological factors during the first three months of a first neck pain episode. The figure shows the development of anxiety, depression, fear avoidance and pain locus of control during the first three months of a first episode of acute neck pain (mean ± standard deviation). * *p* ≤ 0.05. ** *p* ≤ 0.01. *** *p* ≤ 0.001

All tested psychological parameters showed significant reduction within the first month after onset of treatment, but only the parameter ‘depression’ further improved afterwards (Fig. 1). Anxiety (F(2.70,169.78) = 33.54; *p* < 0.001) significantly decreased from baseline to 1 week (*p* = 0.009) and from 1 week to 1 month (*p* = 0.001). Depression (F(2.37,149.44) = 17.25; *p* < 0.001) significantly declined between baseline and 1 week (*p* = 0.022) and 1 month to 3 months (*p* = 0.025). Fear avoidance (F(2.58,146.96) = 25.43; *p* < 0.001) and pain locus of control (F(2.75,167.77) = 17.15; *p* < 0.001) showed a significant reduction from 1 week to 1 month after onset of treatment (*p* < 0.001 and *p* = 0.001, respectively).

The regression model with the psychological factors at each time point as independent variables and the concurrent self-reported outcome as dependent variable explained an increasing proportion of data variability up to 3 months. At 1 and 3 months after the first consultation, high scores in the parameter ‘anxiety’ were concurrent with poor self-reported outcome. The models showed good accuracy for discrimination between improved and unimproved patients at 1 month (AUC = 0.85) and excellent accuracy at 3 months (AUC = 0.98) (Table 2).

The psychological factors at baseline had no influence on the self-reported outcome at 1 week and 1 month. In the model that included only the psychological variables, high level of anxiety at baseline was somewhat predictive for favorable outcome at 1 month (AUC = 0.76). However, anxiety level at baseline became insignificant in the model that included age and baseline pain, where higher age emerged as a predictor for poor recovery (AUC = 0.74) (Table 3). Conversely, depression at baseline emerged from both models as a significant predictor for poor outcome at 3 months (AUC = 0.83 and 0.80, respectively). Pain at baseline could not predict outcome at any point in time.

A high reduction in anxiety between 1 week and 1 month after the first consultation was linked to a significantly higher chance for self-reported improvement at 1 month in both models (Table 4) and the models showed excellent discrimination accuracy (AUC = 0.97 and 0.92, respectively). Self-reported improvement at 3 months was not related to changes in any of the psychological variables.

## Discussion

All psychological parameters that were investigated in this study improved during the first month. Depression declined only somewhat during the first week, but was the only parameter that still improved after the first month. Poor outcome at 1 and 3 months went along with high levels of anxiety. High baseline anxiety was not a risk factor for poor outcome, but its reduction during the first month was highly related to favorable recovery. In contrast, high level of depression at baseline was fairly related to poor recovery at 3 months.

In order to prevent an acute neck pain episode from developing into a chronic problem, the reduction of anxiety at the beginning seems to be a key point in treatment even in this sample of patients who had not previously experienced neck pain. For recovery at 3 months, anxiety was of minor importance, but the factor depression became meaningful. Linton brought up in his review the temporal aspect of the influence of psychological factors on pain and hypothesized that different factors might be relevant at different time points in the course of neck and back pain [7]. The findings of the present study support this developmental approach and bring the patient management in the early phase of treatment into focus. High anxiety at baseline per se does not seem to hinder recovery, provided that it decreases early in the course of treatment. A significant correlation between the reduction in anxiety and in somatic complaints was also reported in a study with orthopedic patients in a rehabilitation setting [24]. This finding suggests that the clinicians should also focus on the changes in the psychological parameters rather than only on their levels at baseline. It further stresses that also in patients with acute neck pain, the multidimensional approach of pain management should be present from the onset of treatment in order to improve outcome, which might not always be the case in daily clinical practice. In the management of acute low back pain, general practitioners were reported to understand pain as a direct representation of tissue injury, and therefore, assessment or management of attitudes and beliefs was of low priority [25]. However, it is well known that appropriate information and the patient’s understanding of pain is crucial in the treatment of acute LBP [26]. A recent qualitative study on attitudes and beliefs of LBP patients found that most participants felt depressed by their pain [27]. This might in most cases not lead into severe depression, which should, in case, promptly be evaluated by a psychiatrist. Nevertheless, the clinicians should be aware that this might result in an attention bias of the patient towards negative information indicating that threatening information might be particularly harmful [27]. Thus, patients with acute non-specific neck pain might benefit from adequate information and communication that targets at reducing anxiety by encouraging self-management of the problem. Cognitive-behavioral therapy (CBT) that focuses on improving coping strategies by diminishing negative thoughts is such an approach. A study that compared usual physiotherapy with a short hands-off intervention by specifically trained physiotherapists using CBT principles could not detect any difference in the effects on sub-acute and chronic neck pain [28]. Similarly, a recent Cochrane Review reported no beneficial effects of CBT for patients with chronic neck pain. For patients with sub-acute neck pain, however, this review found a significantly higher pain reduction at short-term by CBT compared to other interventions [29]. No study was found that investigated the effects of CBT in the management of acute neck pain. The results of the present study, however, encourage the application of CBT principles before neck pain turns into sub-acute or chronic pain. With a view to medication, most pharmacological studies focus on LBP. Non-steroidal anti-inflammatory drugs and muscle relaxants were reported to be effective in acute LBP [30], while the adjunction of an anxiolytic medication (antihistamine) to morphine analgesia did not show an additional benefit in the acute phase of LBP [31].

Anxiety and depression emerged from this study as the most important psychological factors for self-perceived recovery in the first 3 months of a first episode of acute neck pain. Accordingly, the study by Bot et al. on patients with acute neck and shoulder complaints reported that less vitality, which might be seen as a symptom of a depressive disorder, was related to poor recovery after 3 months [12]. Contrary to that study, however, pain at baseline was not a predictor for outcome in the present study. Also fear-avoidance did not emerge as risk factor for poor prognosis for acute neck pain, but was reported to hinder recovery in patients with sub-acute neck pain [4]. In chronic patients, in turn, anxiety and depression were the two main factors related to pain and disability [5]. These findings might partly be explained by the temporal aspect of the psychological variables, but also by the variety of outcomes that were used in these studies. The studies on acute neck pain [12, 13] patients assessed recovery by simple, non–validated questions, while the majority of studies on the role of psychological factors on the course of neck pain used pain, disability or work status as outcome variables [7]. The PGIC as a single-item overall assessment has recently been shown to reflect different specific domains to different degrees. In chronic patients, the overall PGIC particularly reflected improvements in physical activities and mood, rather than improvements in pain and social functioning [32]. Thus, the patient’s impression of change might not linearly influence e.g. socioeconomic relevant variables. Therefore, the impact of anxiety and its reduction in the early phase of acute neck pain on other parameters, such as e.g. sick leave, needs further investigation.

The proportion of patients who reported clinically significant improvement was high (87 %), but well comparable with the numbers in the literature (5 to 10 % of neck problems become chronic [4]). However, it was much higher than reported in a comparable study by Bot et al. [12], which might be explained by differences in the study design: In the latter study, the patients were simply asked whether their symptoms still bothered them. Consequently, patients who answered in the present study that they felt much better or better compared to the beginning, could still have been bothered by their symptoms, which might be the reason for the better outcome in the present study. Nevertheless, the observed proportion of patients with improvement was still small compared to a sample of patients with various acute problems, of whom 97 % reported significant improvement after physiotherapy [33]. This result reflects the persistency of neck pain [3] and emphasizes the need for early attempts to prevent an acute neck pain from transition to a chronic problem.

The strength of this study was that its data design allowed for analyzing not only the influence of psychological factors at certain time points, but also their development over time. Furthermore, the rather rigid inclusion criteria provided a homogeneous patient population and reduced bias resulting from symptom duration and previous pain episodes. However, of course, there were also several limitations. The major limitation of this study was the small number of patients that did not report improvement, which reduced power and might have hidden some findings. This limits generalizability of the results and implies that a confirmation of the results is needed. A second limitation was that the study did not control for the number and type of treatment that the patients underwent during the timespan when they were followed. However, the goal of this study was not to attribute the observed outcome to a certain treatment. The collected data rather reflect clinical practice, where the patients undergo individual treatment according to their needs. In addition, particularly in this sample of acute patients, the observed improvement might at least partly be attributed to natural history. Furthermore, this study did not assess information about other musculoskeletal complaints, although this is a known predictor for an unfavorable course of neck pain [14]. Lastly, the BQ is a valid and reliable questionnaire that reflects the multidimensionality of musculoskeletal pain [18, 20]. Its large advantage is its shortness that allows its use in routine clinical practice. Its items were validated with their counterpart established measures. Nevertheless, the BQ might not be capable of assessing psychological factors in-depth. Thus, the results of this study need to be confirmed by future investigations on the importance of psychological factors in acute non-specific neck pain using separate questionnaires that assess anxiety and depression in more detail. These studies should include a larger number of patients with poor recovery and might investigate strategies to reduce anxiety and depression in the acute phase of neck pain, such as the application of CBT principles by specifically trained health professionals.

## Conclusion

Psychological factors emerged from this study as relevant in the early phase of treatment of patients with a first episode of acute non-specific neck pain. A temporal development of these factors and their influence on self-reported outcome could be observed. Persisting anxiety in the early phase of an acute neck pain problem and depression at baseline emerged as risk factors for poor self-reported recovery and might thus contribute to the transition from acute to chronic pain. The clinical message of this study is that acute neck pain should be regarded as multidimensional. Clinicians should be aware that baseline depression and persisting anxiety might be risk factors for poor prognosis, which should be addressed in the early management of patients with acute non-specific neck pain.
